# Metabolic Risk-attributable Burden of Peripheral Arterial Disease across Socioeconomic Regions: Insights from the Global Burden of Disease Study 2021

**DOI:** 10.2174/011573403X384355250731031837

**Published:** 2025-08-01

**Authors:** Chang Sheng, Shen Chen, Pu Yang, Wei Wang

**Affiliations:** 1 Department of General and Vascular Surgery, Xiangya Hospital, Central South University, Changsha 410008, Hunan, China;; 2 National Clinical Research Center for Geriatric Disorders, Xiangya Hospital, Central South University, Changsha 410008, Hunan, China;; 3 Clinical Research Center for Vascular Intervention in Hunan Province, Xiangya Hospital, Central South University, Changsha 410008, Hunan, China

**Keywords:** Peripheral arterial disease, global burden of disease, metabolic risks, socioeconomic level, socio-demographic index, age-standardized mortality rates

## Abstract

**Introduction:**

Peripheral arterial disease (PAD) is a significant contributor to global morbidity, with regional burdens exhibiting considerable heterogeneity. The PAD burden attributable to metabolic risks across regions with varying socioeconomic levels has yet to be adequately characterized.

**Methods:**

This study analyzes PAD burden attributable to metabolic risks across different socioeconomic regions using data from the Global Burden of Disease (GBD) 2021 study. We analyzed data on PAD attributable to metabolic risks, including high systolic blood pressure (SBP), high fasting plasma glucose (FPG), kidney dysfunction (KD), and high body mass index (BMI), across four health systems, four world bank income levels, five socio-demographic index (SDI) levels, and 21 GBD regions, from 1990 to 2021. We presented age-standardized mortality rates (ASMR), age-standardized disability-adjusted life year rates (ASDR) and estimated annual percentage changes (EAPC) to assess burden and trends.

**Results:**

In 2021, the burden of PAD due to metabolic risks remained high in regions with higher socioeconomic levels, though it showed a declining trend. Conversely, the burden in regions with lower socioeconomic levels was also high but exhibited an increasing trend. High FPG has become a significant factor in the burden of PAD, particularly in higher socioeconomic regions. Gender disparities in the burden of PAD attributable to metabolic risks were evident, with males exhibiting higher ASMR and ASDR, although females in middle-income regions had slightly elevated ASDRs. Finally, an inverted “U” relationship was observed between SDI and burden, with regions around an SDI of 0.75 exhibiting a higher burden of PAD attributable to metabolic risks.

**Discussion:**

These findings underscore the urgent need to tailor region-specific public health strategies that account for socioeconomic disparities in metabolic risk exposures contributing to the PAD burden.

**Conclusions:**

Effective public health interventions targeting these metabolic risks are urgently needed, especially in low-socioeconomic regions where the burden remains disproportionately high. Enhanced blood glucose control and early intervention strategies should be prioritized to mitigate the growing impact of PAD globally.

## 1 INTRODUCTION

Lower extremity peripheral arterial disease (PAD) is an atherosclerotic condition characterized by the narrowing or occlusion of arteries in the lower limbs, leading to symptoms such as intermittent claudication, ischemic pain, and functional impairment [1]. In 2021, PAD accounted for 1.56 million (95% uncertainty intervals [UI] 1.27 to 2.05) Disability-Adjusted Life Years (DALYs). That same year, there were 56.6 million (95% UI 47.9 to 67.7) prevalent cases of PAD among individuals aged 40–70, and 57.1 million (95% UI 48.6 to 67.9) prevalent cases in those over 70 [2].

Data from 2019 reveal that age-standardized mortality rates and DALYs related to PAD have shown minimal variation over time [3]. But, the burden of PAD-related diseases has been underestimated, with significant heterogeneity across gender, age, and socioeconomic factors [4]. PAD prevalence is greater in nations with a high income and Socio-demographic Index (SDI), but the DALY rates in low income and SDI countries are disproportionately high relative to prevalence. This suggests that PAD has not been adequately managed in these regions [5]. Populations in low socioeconomic regions are also typically more exposed to cardiovascular hazards such as hyperglycemia [6], and hypertension [7]. The prevalence and burden of cardiovascular diseases are rising continuously in middle-income and low- countries, likely driven by industrialization, urbanization, and the increasing prevalence of metabolic risk factors [8]. In 2021, the top five contributors to PAD-related DALYs were high fasting plasma glucose (FPG), kidney dysfunction (KD), smoking, high body mass index (BMI), and high systolic blood pressure (SBP), with four of these classified as metabolic risks 2. Approximately 70% of the global PAD burden can be attributed to alterable risk factors. Global health interventions could reduce the PAD burden by targeting modifiable risk factors. Therefore, it is crucial to examine the global landscape of PAD associated with metabolic risks, taking into account various sociodemographic and spatiotemporal trends.

This study aims to provide comprehensive estimates of disability and mortality linked to the global burden of PAD due to metabolic risks, utilizing data from the Global Burden of Disease (GBD), Injuries, and Risk Factors Study 2021.

## 2 MATERIALS AND METHODS

### 2.1 Data Source

This study on Peripheral Artery Disease (PAD) utilizes data from the GBD 2021, which offers comprehensive epidemiological estimates for 371 diseases and injuries from 1990 to 2021 across 21 regions and 204 countries and territories [9].

The data can be accessed freely *via* the Global Health Data Exchange (https://ghdx.healthdata.org/gbd-2021/sources). The GBD study compiles disease incidence and prevalence by integrating diverse data sources, including population-representative studies identified through systematic literature reviews, cohort analyses, and scientific reports from national registries, as well as data from population surveys and health system administrative records. To address global data quality variability, a Bayesian metaregression tool called DisMod 2.1 is utilized to adjust for under-reporting and inconsistencies, allowing for standardized estimates that are comparable across different regions worldwide. Detailed information on the data sources, methodologies, and statistical modeling is available in prior reports [9, 10]. For this study, we downloaded age-specific data related to PAD attributable to metabolic risks across 21 regions, 4 health systems, 4 World Bank income levels, and 5 SDI classifications from 1990 to 2021. This data set includes the number of DALYs, deaths, age-standardized disability-adjusted life years rate (ASDR), and age-standardized mortality rate (ASMR). Additionally, the database provides information on four metabolic risk factors contributing to PAD burden: high SBP, high FPG, KD, and high BMI. The data were accessed on August 2, 2024, and ethics approval was unnecessary as the study used publicly available data without involving private information (Supplementary Table **S1**).

### 2.2 Definitions

PAD is characterized by an ankle-brachial index (ABI) < 0.90. It commonly presents with intermittent claudication, which is defined by pain in the legs during physical activity in individuals with an ABI below this threshold. Detailed International Classification of Diseases (ICD) codes for PAD are provided in Supplementary Table **S2**. In the GBD 2021 study, high SBP is defined as exceeding 105–115 mmHg, which represents the theoretical minimum risk exposure level (TMREL) that reduces population-level risk. Elevated FPG is defined as serum glucose levels > 4.9–5.3 mmol/L. KD is characterized by an estimated glomerular filtration rate (eGFR) < 60 ml/min/1.73 m^2^ or an albumin-to-creatinine ratio ≥ 30 mg/g, with TMREL values being an albumin-to-creatinine ratio < 30 mg/g and an eGFR ≥ 60 ml/min/1.73 m^2^. For adults (age 20+), high BMI is defined > 20–23 kg/m^2^. The SDI is a composite measure reflecting development status, which is strongly correlated with health outcomes, and is calculated based on per capita income, educational attainment, and fertility rates. The SDI was developed by the Institute for Health Metrics and Evaluation (IHME) at the University of Washington and was first used in the GBD 2015 study [11]. The GBD Study categorizes regions and countries by socioeconomic development, including classifications by health systems (minimal, limited, basic, advanced), SDI regions (low, low-middle, middle, high-middle, high), and World Bank income levels (low, low-middle, upper-middle, high).

### 2.3 Disability-adjusted Life Years

DALYs are an established indicator for evaluating disease burden, representing the total years of healthy life lost due to a disease, from onset to death. This measure combines years of life lost (YLLs) due to premature mortality and years lived with disability (YLDs) into a single metric, expressed by the formula:

𝐷𝐴𝐿𝑌𝑠 = 𝑌𝐿𝐿𝑠 + 𝑌𝐿𝐷𝑠

ASDR and ASMR are presented per one hundred thousand individuals, categorized by age, gender, metabolic risks, geographical location, and socioeconomic development level. To calculate the percentage change in these rates from 2010 to 2021, the following formula is used:

𝑝𝑒𝑟𝑐𝑒𝑛𝑡𝑎𝑔𝑒 𝑐ℎ𝑎𝑔𝑛𝑒 = (2021𝑟𝑎𝑡𝑒𝑠 - 2010𝑟𝑎𝑡𝑒𝑠)/2010𝑟𝑎𝑡𝑒𝑠

### 2.4 Estimated Annual Percentage Change and Percentage Change

The estimated annual percentage change (EAPC) is a commonly employed measure for monitoring trends in epidemiological metrics, including prevalence and incidence rates, over a period [12-15]. An age-standardized rate (ASR) is deemed to be on an upward trend if the lower limit of its 95% confidence intervals (CI) is above 0. Conversely, if the upper limit of its 95% CI is below 0, the ASR is declining. EAPC is calculated by fitting the natural logarithm of the rate into a linear regression model, where time serves as the independent variable. The formula used for this calculation is:

𝑦 = 𝛼 + 𝛽𝑥 + ε

𝐸𝐴𝑃𝐶 = 100 x (exp (𝛽) - 1)

In this context, 𝑥 denotes the year, 𝑦 signifies the natural logarithm of rates (such as prevalence or incidence), 𝛼 represents the intercept, 𝛽 indicates the slope, and 𝜖 stands for random error. The 95% CI for the EAPC is calculated based on this model.

In addition, relationships among ASMR, ASDR, and SDI were examined through locally estimated scatterplot smoothing (LOESS) regression across 21 GBD regions. Data cleaning, computations, and graph generation were conducted using R software (version 4.3.3). Visualizations were developed with the ggplot2 package, and final formatting was accomplished using Adobe Illustrator (version 26.0).

## 3 RESULTS

### 3.1 Overview of Metabolic Risk-attributable Burden of Peripheral Arterial Disease in 2021

The metabolic risk-attributable burden of PAD, including PAD deaths, ASMR, DALYs, and ASDR in four health systems in 2021, is presented in Table **1** and Supplementary Table **S3**. Among these health systems, the ASMR for PAD attributable to metabolic risks was highest in the Advanced Health System, at 0.94 (95% UI 0.78 to 1.09) per 100,000 population (Table **1**). Similarly, the ASDR reached 18.43 (95% UI 15.24 to 22.86) per 100,000 population (Supplementary Table **S3**). Notably, within each health system, PAD attributable to high FPG posed a greater burden compared to other metabolic risks, with the Advanced Health System exhibiting the greatest ASMR at 0.57 (95% UI 0.45 to 0.69) per 100,000 and the greatest ASDR at 11.21 (95% UI 8.76 to 14.43) per 100,000.

While the overall burden of PAD attributable to metabolic risks was highest in the Advanced Health System, World Bank High-Income, and High SDI regions, the ASMR and ASDR were also elevated in the Minimal Health System compared to the Limited and Basic Health Systems, with values of 0.69 (95% UI 0.31 to 1.35) and 13.56 (95% UI 6.81 to 23.84), respectively. This pattern was consistent across all four metabolic risks. Similar trends were observed across income regions and SDI regions, as detailed in Tables **2** and **3**, Supplementary Tables **S4** and **S5**. The burden of PAD attributable to metabolic risks is also visually represented in Supplementary Fig. (**S1**).

The burden of PAD deaths and ASMR, as well as DALYs and ASDR attributable to metabolic risks in the 21 GBD regions in 2021, is provided in Supplementary Tables **S6** and **S7**. Specifically, the highest ASMR of PAD attributable to metabolic risks was recorded in Central Europe (1.68 per 100,000, 95% UI 1.38 to 2.00), followed by Eastern Europe (1.54 per 100,000, 95% UI 1.24 to 1.85) and Southern Sub-Saharan Africa (1.37 per 100,000, 95% UI 1.17 to 1.62). The highest ASDR was observed in Eastern Europe (29.05 per 100,000, 95% UI 23.46 to 35.15), followed by Southern Sub-Saharan Africa (28.88 per 100,000, 95% UI 24.2 to 35.14) and Central Europe (28.65 per 100,000, 95% UI 23.78 to 34.24). Comparing the ASMR and ASDR across all GBD regions, Oceania ranks higher in ASDR (6.50 per 100,000, 95% UI 3.74 to 11.30) but lower in ASMR (0.09 per 100,000, 95% UI 0.05 to 0.13). This may indicate a need for improved care and treatment strategies in the region.

### 3.2 Gender and Age Patterns of Metabolic Risk-Attri-butable Burden of Peripheral Arterial Disease in 2021

In all four health systems, males exhibited higher ASMR for PAD attributable to metabolic risks than females, with the highest ASMR observed in males within the Advanced Health System, at 1.16 (95% UI 0.98 to 1.32) per 100,000 population (Supplementary Fig. **S2**). Similarly, the greatest ASDR in males was also in the Advanced Health System, at 22.44 (95% UI 18.95 to 26.94) per 100,000 population. Notably, in the Basic Health System, females exhibited a higher ASDR for PAD attributable to metabolic risks (6.81 per 100,000, 95% UI 4.46 to 11.25) than males (5.90 per 100,000, 95% UI 4.52 to 8.01), a trend observed across all four metabolic risks. Similar gender patterns were observed across income regions and SDI regions, as illustrated in (Supplementary Figs. **S3** and **S4**).

Across various socioeconomic classifications, the burden of PAD attributable to metabolic risks showed an increasing trend with age (Supplementary Figs. **S5-S7**). The highest ASMR for PAD attributable to metabolic risks in the 50-69 age group was observed in the Advanced Health System (1.15 per 100,000, 95% UI 0.96 to 1.32), World Bank High Income (0.96 per 100,000, 95% UI 0.82 to 1.09), and High SDI regions (0.88 per 100,000, 95% UI 0.76 to 1.00). Similarly, the highest ASDR in this age group was found in the Advanced Health System (40.49 per 100,000, 95% UI 32.89 to 51.32), World Bank High Income (35.84 per 100,000, 95% UI 28.79 to 46.92), and High SDI regions (33.65 per 100,000, 95% UI 26.92 to 44.39). Notably, a decline in PAD rates attributable to high FPG was observed after the 90-94 age group in regions such as the Minimal Health System, Limited Health System, World Bank Low Income, World Bank Lower Middle Income, Low SDI, and Low-middle SDI. More detailed data are available online (https://vizhub.healthdata.org/gbd-results/).

### 3.3 Trend of Metabolic Risk-attributable Burden of Peripheral Arterial Disease

**Fig. (1):**
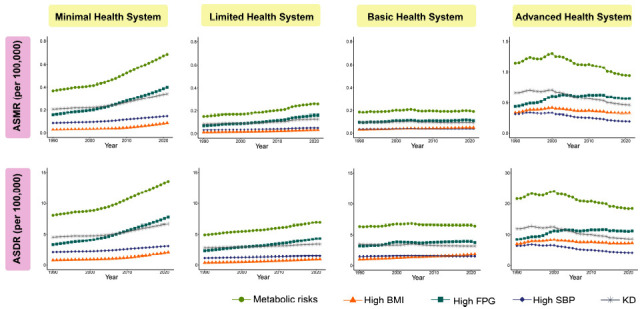
Trends in peripheral artery disease attributable to metabolic risks across different health systems: deaths and disability-adjusted life years from 1990 to 2021. **Abbreviations:** ASMR: age-standardized mortality rate; ASDR: age-standardized disability-adjusted life years rate; DALYs: disability-adjusted life years; BMI: body-mass index; FPG: fasting plasma glucose; SBP: systolic blood pressure; KD: kidney dysfunction.

**Fig. (2):**
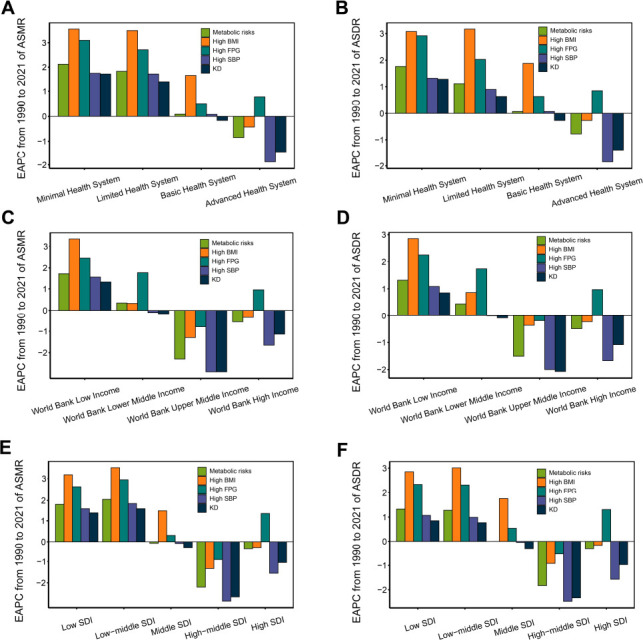
Estimated annual percentage change in age-standardized mortality rate and age-standardized disability-adjusted life years rate attributable to metabolic risks for peripheral artery disease across different regions (1990–2021). A) Estimated annual percentage change in ASMR attributable to metabolic risks for peripheral artery disease across different health systems (1990–2021). B) Estimated annual percentage change in ASDR attributable to metabolic risks for peripheral artery disease across different health systems (1990–2021). C) Estimated annual percentage change in ASMR attributable to metabolic risks for peripheral artery disease across different income regions (1990–2021). D) Estimated annual percentage change in ASDR attributable to metabolic risks for peripheral artery disease across different income regions (1990–2021). E) Estimated annual percentage change in ASMR attributable to metabolic risks for peripheral artery disease across different SDI regions (1990–2021). F) Estimated annual percentage change in ASDR attributable to metabolic risks for peripheral artery disease across different SDI regions (1990–2021). **Abbreviations:** SDI: socio-demographic index; EAPC: estimated annual percentage change; ASMR: age-standardized mortality rate; ASDR: age-standardized disability-adjusted life years rate; BMI: body-mass index; FPG: fasting plasma glucose; SBP: systolic blood pressure; KD: kidney dysfunction.

In the Minimal Health System, the ASMR for PAD attributable to metabolic risks rose from 1990 to 2021, showing an EAPC of 2.12 (95% CI: 1.98 to 2.25). The most notable rise was detected in ASMR attributable to high BMI, showing an EAPC of 3.55 (95% CI: 3.22 to 3.88) (Figs. **1** and **2**, and Table **4**). Similarly, the ASDR for PAD attributable to metabolic risks also increased during this time frame (EAPC: 1.76 [95% CI: 1.63 to 1.88]). In the Limited Health System, the upward trend was weaker, showing an EAPC of 1.83 (95% CI: 1.70 to 1.96) for ASMR and 1.11 (95% CI: 1.07 to 1.15) for ASDR. In contrast, minimal changes were observed in the Basic Health System. Conversely, in the Advanced Health System, both ASMR and ASDR for PAD attributable to metabolic risks showed a decreasing trend from 1990 to 2021, with EAPCs of -0.87 (95% CI: -1.07 to -0.67) for ASMR and -0.78 (95% CI: -0.94 to -0.62) for ASDR. However, the burden attributable to high FPG enhanced, showing an EAPC of 0.79 (95% CI: 0.47 to 1.11) for ASMR and 0.85 (95% CI: 0.60 to 1.11) for ASDR.

**Fig. (3):**
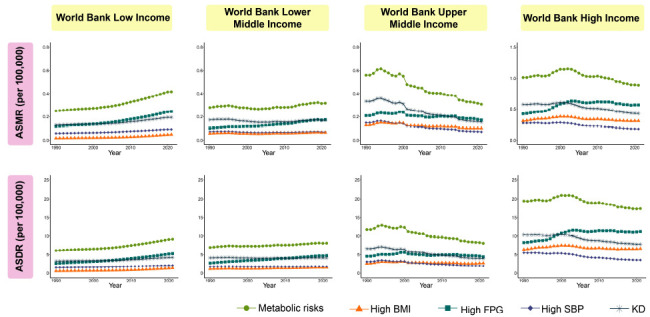
Trends in peripheral artery disease attributable to metabolic risks across different income regions: deaths and disability-adjusted life years from 1990 to 2021. **Abbreviation:** ASMR: age-standardized mortality rate; ASDR: age-standardized disability-adjusted life years rate; DALYs: disability-adjusted life years; BMI: body-mass index; FPG: fasting plasma glucose; SBP: systolic blood pressure; KD: kidney dysfunction.

**Fig. (4):**
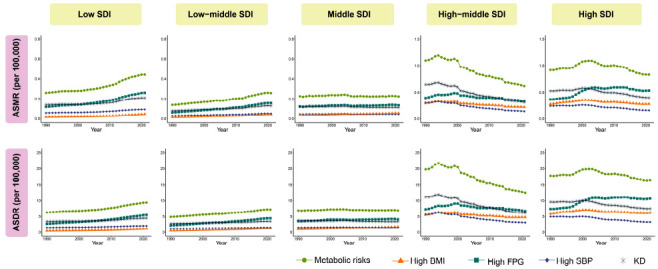
Trends in peripheral artery disease attributable to metabolic risks across different SDI regions: deaths and disability-adjusted life years from 1990 to 2021. **Abbreviations:** SDI: socio-demographic index; ASMR: age-standardized mortality rate; ASDR: age-standardized disability-adjusted life years rate; DALYs: disability-adjusted life years; BMI: body-mass index; FPG: fasting plasma glucose; SBP: systolic blood pressure; KD: kidney dysfunction.

Trends in the World Bank High-Income group were like those in the Advanced Health System. However, in the World Bank Upper Middle-Income group, a consistent decrease in PAD burden attributable to metabolic risks was observed across the four metabolic risks (Figs. **2** and **3** and Table **5**). High SDI regions showed trends similar to those in the Advanced Health System, while Middle SDI and the Basic Health System exhibited minimal changes over the 30-year period, with the exception of an increasing trend in the burden of PAD attributable to elevated BMI (Figs. **2** and **4** and Table **6**).

To assess recent changes in PAD burden, we calculated the percentage change in PAD attributable to metabolic risks across different regions from 2010 to 2021 (Fig. **2**). In the Minimal Health System, the ASMR for PAD attributable to metabolic risks increased by 31.3% (95% CI: 8.6% to 71.7%), with the most significant increase in ASMR attributable to high BMI (82.8% [95% CI: 40.2% to 155.1%]) (Supplementary Table **S8**). Correspondingly, the ASDR for PAD attributable to metabolic risks also increased by 27.0% (95% CI: 8.8% to 52.9%). In contrast, the Advanced Health System showed a percentage decrease in PAD burden, with an ASMR reduction of -16.6% (95% CI: -20.0% to -12.7%) and an ASDR reduction of -11.3% (95% CI: -15.4% to -6.9%). In the Basic Health System, although the overall percentage change in PAD burden was negative, the burden attributable to high BMI increased, with ASMR rising by 15.1% (95% CI: 8.1% to 24.7%) and ASDR rising by 21.9% (95% CI: 15.4% to 29.5%). Detailed findings for income regions and SDI regions are available in Supplementary Tables **S9** and **S10**. Notably, among GBD regions, the highest percentage increase in PAD burden attributable to metabolic risks was identified in Andean Latin America (54.2% [95% CI: 24.9% to 91.1%]), while the most significant decrease was seen in Western Europe (-26.9% [95% CI: -30.6% to -22.6%]) (Supplementary Tables **S11** and **S12**).

### 3.4 Metabolic Risk-attributable Burden of Peripheral Arterial Disease and SDI

**Fig. (5):**
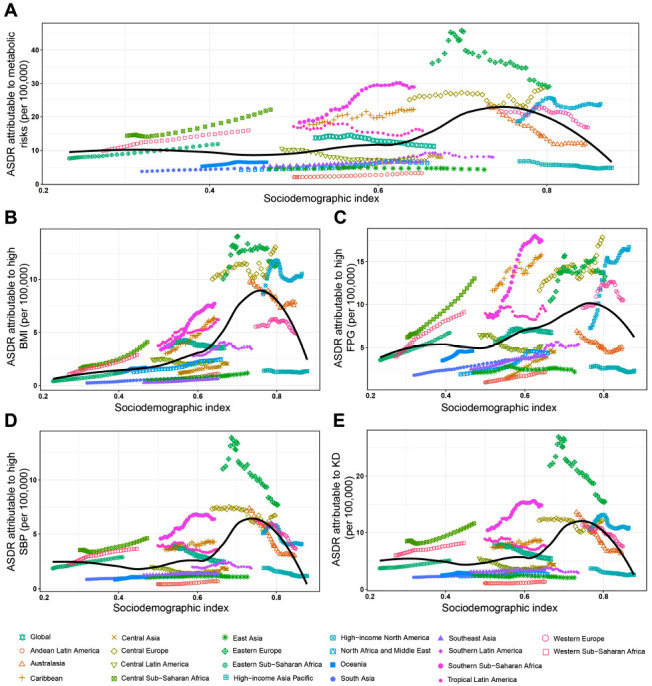
Relationship between metabolic risk-attributable age-standardized disability-adjusted life years rate of peripheral arterial disease and socio-demographic index Across 21 GBD Regions, 1990–2021. **A**) ASDR of peripheral artery disease attributable to metabolic risks for the 21 global burden of disease regions by sociodemographic index, 1990–2021. **B**) ASDR of peripheral artery disease attributable to high BMI for the 21 global burden of disease regions by sociodemographic index, 1990–2021. **C**) ASDR of peripheral artery disease attributable to high FPG for the 21 global burden of disease regions by sociodemographic index, 1990–2021. **D**) ASDR of peripheral artery disease attributable to high SBP for the 21 global burden of disease regions by sociodemographic index, 1990–2021. **E**) ASDR of peripheral artery disease attributable to kidney dysfunction for the 21 global burden of disease regions by sociodemographic index, 1990–2021. Thirty-two points are plotted for each region and show the observed ASDR from 1990 to 2021 for that region. Expected values, based on sociodemographic index and disease rates in all locations, are shown as a solid line. Regions above the solid line represent a higher-than-expected burden and regions below the line show a lower-than-expected burden. Abbreviation: SDI: socio-demographic index; ASDR: age-standardized disability-adjusted life years rate; BMI: body-mass index; FPG: fasting plasma glucose; SBP: systolic blood pressure; KD: kidney dysfunction.

The study observed an inverted U-shaped link between regional SDI and ASMR/ASDR attributable to metabolic risks (Fig. **5** and Supplementary Fig. **S8**) from 1990 to 2021. The highest PAD burden was in regions with an SDI around 0.75. Most GBD regions showed increasing trends in ASMR and ASDR, while a few regions demonstrated a decreasing trend. Among all regions, Eastern Europe recorded the highest ASMR and ASDR for metabolic risks, whereas Western Europe reported the lowest. When focusing on ASMR/ASDR attributable to high FPG, Southern Sub-Saharan Africa, Central Europe, and High-Income North America ranked higher among GBD regions, and the overall trend is increasing.

## 4 DISCUSSION

In this study, we assess nearly 30 years of deaths and DALYs burden from PAD linked to metabolic risks across various socioeconomic regions, with a focus on the results from 2021 and the trends. The burden of PAD shows significant regional variation, reflecting the impact of different socioeconomic classifications. We, therefore, present the burden of PAD attributable to metabolic risks across various classifications and GBD regions. Furthermore, we analyzed the correlation between SDI and the burden of PAD attributable to metabolic risks over the past 30 years across 21 GBD regions.

The global burden of PAD attributable to metabolic risks has not been adequately addressed. This burden has increased in regions with lower socioeconomic levels, while in regions with higher socioeconomic levels, it remains high despite a decreasing trend [3, 16]. Similar to previously observed patterns, the burden of PAD attributable to metabolic risks in 2021 was notably high in regions with lower socioeconomic levels, suggesting that inadequate management of metabolic risks may underlie the higher PAD burden in these areas [5]. We also consistently found that the burden from peripheral artery disease attributable to high FPG is predominant across regions with different socioeconomic levels, which is consistent with previous studies [4, 12]. Of particular concern is the significant and rising burden of PAD attributable to high FPG, even in high socioeconomic regions. Over the past 30 years, the ASDR for diabetes has increased globally by 24.4%, which may contribute to a higher proportion of PAD cases related to diabetes [17]. Diabetic patients with PAD tend to die at a younger age and have a higher mortality rate compared to those without diabetes [18]. Additionally, PAD patients with diabetes face a significantly increased risk of future amputations [19, 20]. This underscores the critical importance of prioritizing blood glucose control and diabetes management. Patients with diabetes should be considered at high risk for PAD, warranting vigilant monitoring and early intervention 1. Recent trends in diabetes management, including improved glucose control through newer medications (*e.g.*, SGLT2 inhibitors and GLP-1 receptor agonists) and more integrated care approaches, may help reduce the overall burden of PAD. However, despite advancements in diabetes management, the burden of PAD remains high in many high socioeconomic regions, suggesting a need for more integrated management strategies that address both metabolic risks and PAD simultaneously. Enhanced collaboration between diabetes care providers and vascular specialists could lead to earlier diagnosis and treatment of PAD, ultimately reducing its burden in these regions.

To further explore potential causes, we analyzed the correlation between the SDI and the burden of PAD due to metabolic risks over the past 30 years across various GBD regions. This relationship exhibits an inverted U-shaped curve, where the burden increases with rising SDI in lower SDI regions but decreases as SDI continues to rise in higher SDI regions. Notably, in some higher SDI regions, we observed an increasing burden attributable to high FPG, even as overall PAD burden decreased. This study highlights the critical role of metabolic risk factors in shaping the PAD burden across different socioeconomic regions, informing the development of targeted prevention strategies.

Our findings reveal that although metabolic risks are modifiable, the burden from PAD attributable to these risks has not been effectively managed in low- to middle-socioeconomic regions over the past 30 years. It is reasonable to attribute this similarity to the fact that metabolic risks are the primary contributing factors to the PAD burden [5]. The inadequate control of PAD burden attributable to metabolic risks may be attributed to several factors: (1) Awareness of PAD remains low, with only 25.8% of the population being aware of the condition, compared to higher awareness for stroke and coronary artery disease [21]; (2) The diagnostic rate for PAD is low, as PAD often remains asymptomatic and undiagnosed until advanced stages [22]; (3) Inadequate early treatment of PAD. Previous research has shown that in developed countries, nearly 50% of PAD patients do not receive appropriate antiplatelet and lipid-lowering therapy [23-25]. Moreover, the number of PAD patients receiving proper treatment in developing countries is significantly lower than in developed countries [26]. Current PAD management guidelines focus primarily on symptomatic PAD, potentially delaying the diagnosis and treatment of early-stage or high-risk patients [1]. These factors likely contribute to insufficient and delayed management of PAD in many regions. However, our findings also show a decrease in PAD burden in higher socioeconomic regions, where effective interventions, such as blood pressure reduction, have proven beneficial. These interventions should be adapted and implemented in regions with lower socioeconomic levels. For instance, in high-SDI regions, multifaceted intervention programs combining hypertension and diabetes management, smoking cessation, and public awareness campaigns have demonstrated effectiveness in reducing PAD burden. One example is the WHO Package of Essential Noncommunicable (PEN) Disease Interventions, which has been adapted in several high-income settings to integrate risk factor screening with lifestyle counseling and improved access to essential medications. These models could be adapted to low-SDI regions by leveraging existing community health infrastructure, such as community health workers, task-shifting strategies, and simplified treatment protocols to improve accessibility and sustainability. Such adaptations would be particularly relevant in resource-limited settings and could potentially yield significant reductions in PAD burden.

Gender differences in PAD burden attributable to metabolic risks are significant across most socioeconomic regions. In general, males bear a higher burden than females, consistent with previous studies indicating that the total DALYs burden caused by modifiable risk factors is higher in males [5]. However, in Basic Health System, World Bank Upper Middle Income, and Middle SDI regions, females have a higher ASDR from PAD attributable to metabolic risks than males. These gender differences may be influenced by several factors. First, differences in healthcare access can affect diagnosis and treatment. In many regions, males may have less frequent healthcare visits or delayed diagnoses, potentially leading to higher disability rates. Second, lifestyle factors such as smoking and physical activity patterns may differ between genders, with males often engaging in higher-risk behaviors, thus contributing to a higher PAD burden. Lastly, biological susceptibility, including genetic and hormonal factors, could also play a role, as men may have a higher predisposition to metabolic diseases such as diabetes and hypertension, which are strongly linked to PAD. Understanding these factors is critical for developing gender-specific interventions aimed at reducing PAD burden, particularly in high-risk male populations. This finding suggests that early PAD screening and timely treatment are particularly important for females in these regions. While previous studies have shown that PAD prevalence is higher in females, particularly in low- and middle-income regions [16, 27]. Our study further supplements this knowledge by highlighting that, within the burden from PAD attributable to metabolic risks, males constitute the majority, even though the prevalence of PAD is higher in females. Our study extends this understanding by revealing that, within the burden from PAD attributable to metabolic risks, males are more likely to suffer from disability, potentially due to later-stage diagnosis or higher complication risks. Addressing metabolic risks in male PAD patients remains a priority.

## STUDY LIMITATIONS

There are some limitations in this study. First, the significant variations in data quality across regions and countries, coupled with inconsistent data completeness, may result in deviations from the actual situation. Nevertheless, to address this limitation, the GBD study adopts a rigorous and standardized modeling framework, thereby minimizing potential biases to a certain extent. Our analysis relies on the quality of the initial GBD study, and we face limitations in conducting further quantitative analyses. Additionally, the study lacks statistical analysis to determine which socioeconomic classification method is most suitable for assessing PAD burden, as our focus was on comparing burden differences across regions. Future studies could explore alternative classification frameworks and assess their comparative performance to identify the most appropriate approach for capturing socioeconomic disparities in PAD burden. Moreover, cultural differences may affect the accuracy of self-reported symptoms of intermittent claudication [28], potentially leading to an underestimate disease burden. Furthermore, the exclusion of hypercholesterolemia from the GBD database restricts analysis of metabolic risk factors contributing to PAD. Another notable limitation is the absence of data on PAD-related leg outcomes, which constrains a more detailed estimation of PAD burden. Future work should consider integrating such limb-specific outcomes to enhance the precision and clinical relevance of PAD burden assessments. Finally, it should be noted that regional variations in PAD diagnosis and treatment practices are not fully captured in the GBD estimates. Integrating case studies from specific countries or health systems—particularly those that have demonstrated success in reducing PAD burden through targeted metabolic risk interventions—would be valuable in further contextualizing and validating our findings.

## CONCLUSION

Over the past three decades, the global burden of peripheral artery disease (PAD) attributable to metabolic risks has remained substantial and persistent, underscoring an urgent need for effective and sustained public health interventions. Policymakers must take into account the specific socioeconomic context when formulating and implementing strategies aimed at preventing and reducing the PAD burden. Socioeconomic disparities influence not only the prevalence of metabolic risk factors but also access to healthcare, early detection, and treatment, which collectively affect disease outcomes. Given that metabolic risks-such as high systolic blood pressure, high fasting plasma glucose (FPG), high body mass index, and kidney dysfunction-account for a significant portion of the global PAD burden, comprehensive and multi-pronged public health measures focusing on the management and control of these modifiable risk factors could lead to considerable reductions in disease incidence and disability. These strategies should include population-wide screening, health education, lifestyle modifications, and improved clinical management, tailored to the needs of specific regions and vulnerable groups. Overall, findings from this GBD 2021 analysis offer valuable insights for guiding evidence-based healthcare planning, prevention, and resource allocation. They highlight the importance of addressing modifiable metabolic risks through integrated, context-specific strategies to reduce the global and regional burden of PAD in an equitable and sustainable manner.

## Figures and Tables

**Table 1:** Death from peripheral artery disease attributable to metabolic risks across different health system in 2021.

Etiology	Minimal Health System		Limited Health System		Basic Health System		Advanced Health System	
-	Number, 2021 (95% UI)	ASMR per 100,000 (95% UI)	Number, 2021 (95% UI)	ASMR per 100,000 (95% UI)	Number, 2021 (95% UI)	ASMR per 100,000 (95% UI)	Number, 2021 (95% UI)	ASMR per 100,000 (95% UI)
Metabolic risks	541 (246, 1022)	0.69 (0.31, 1.35)	3730 (2467, 5594)	0.26 (0.17, 0.39)	5993 (5031, 7020)	0.19 (0.16, 0.22)	32120 (26472, 37201)	0.94 (0.78, 1.09)
High BMI	80 (17, 234.9)	0.09 (0.02, 0.25)	485 (129, 1223)	0.03 (0.01, 0.08)	1704 (487, 4043)	0.05 (0.02, 0.13)	10987 (3187, 25522)	0.33 (0.10, 0.77)
High FPG	314 (132, 615)	0.40 (0.17, 0.80)	2321 (1446, 3619)	0.16 (0.10, 0.25)	3574 (2808, 4314)	0.11 (0.09, 0.14)	19369 (15162, 23393)	0.57 (0.45, 0.69)
High SBP	121 (16, 270)	0.15 (0.02, 0.34)	784 (120, 1558)	0.05 (0.01, 0.11)	1303 (266, 2381)	0.04 (0.01, 0.07)	6668 (1392, 11847)	0.20 (0.04, 0.35)
KD	265 (113, 537)	0.34 (0.15, 0.72)	1786 (1021, 2866)	0.13 (0.07, 0.20)	2925 (1965, 3823)	0.10 (0.06, 0.12)	16108 (10603, 21217)	0.46 (0.31, 0.61)

**Abbreviations:** UI: uncertainty intervals; ASMR: age-standardized mortality rate; BMI: body-mass index; FPG: fasting plasma glucose; SBP: systolic blood pressure; KD: kidney dysfunction.

**Table 2:** Death from peripheral artery disease attributable to metabolic risks across different income regions in 2021.

Etiology	World Bank Low Income		World Bank Lower Middle Income		World Bank Upper Middle Income		World Bank High Income	
-	Number, 2021 (95% UI)	ASMR per 100000 (95% UI)	Number, 2021 (95% UI)	ASMR per 100000 (95% UI)	Number, 2021 (95% UI)	ASMR per 100000 (95% UI)	Number, 2021 (95% UI)	ASMR per 100000 (95% UI)
Metabolic risks	942 (462, 1748)	0.42 (0.21, 0.79)	5628 (4136, 7479)	0.32 (0.23, 0.42)	9725 (8147, 11284)	0.31 (0.25, 0.36)	26089 (21144, 30196)	0.88 (0.73, 1.02)
High BMI	121 (29, 343)	0.05 (0.01, 0.14)	1175 (301, 2909)	0.06 (0.02, 0.16)	3210 (892, 7601)	0.10 (0.03, 0.24)	8749 (2603, 20064)	0.31 (0.09, 0.7)
High FPG	549 (261, 1058)	0.24 (0.11, 0.48)	3148 (2175, 4368)	0.17 (0.12, 0.24)	5459 (4278, 6675)	0.17 (0.13, 0.21)	16423 (12681, 19812)	0.56 (0.44, 0.68)
High SBP	213 (26, 463)	0.09 (0.01, 0.20)	1262 (226, 2347)	0.07 (0.01, 0.13)	2248 (467, 4010)	0.07 (0.01, 0.13)	5152 (1074, 9190)	0.18 (0.04, 0.31)
KD	433 (198, 858)	0.19 (0.09, 0.39)	2919 (1867, 4192)	0.17 (0.11, 0.24)	4827 (3274, 6381)	0.16 (0.11, 0.20)	12906 (8412, 16898)	0.42 (0.28, 0.56)

**Abbreviations:** UI: uncertainty intervals; ASMR: age-standardized mortality rate; BMI: body-mass index; FPG: fasting plasma glucose; SBP: systolic blood pressure; KD: kidney dysfunction.

**Table 3:** Death from peripheral artery disease attributable to metabolic risks across different SDI regions in 2021.

Etiology	Low SDI		Low-middle SDI		Middle SDI		High-middle SDI		High SDI	
-	Number, 2021 (95% UI)	ASMR per 100000 (95% UI)	Number, 2021 (95% UI)	ASMR per 100000 (95% UI)	Number, 2021 (95% UI)	ASMR per 100000 (95% UI)	Number, 2021 (95% UI)	ASMR per 100000 (95% UI)	Number, 2021 (95% UI)	ASMR per 100000 (95% UI)
Metabolic risks	1474 (735, 2775)	0.44 (0.22, 0.84)	2816 (2060, 3786)	0.26 (0.19, 0.35)	4986 (4188, 5803)	0.23 (0.19, 0.26)	11762 (9685, 13753)	0.62 (0.51, 0.72)	21346 (17331, 24625)	0.83 (0.68, 0.95)
High BMI	175 (41, 508)	0.05 (0.01, 0.13)	510 (140, 1215)	0.05 (0.01, 0.11)	1312 (375, 3124)	0.06 (0.02, 0.14)	4278 (1175, 9909)	0.22 (0.06, 0.52)	6980 (2072, 16003)	0.28 (0.08, 0.64)
High FPG	875 (420, 1745)	0.26 (0.12, 0.53)	1737 (1213, 2400)	0.16 (0.11, 0.22)	3036 (2418, 3717)	0.14 (0.11, 0.17)	6348 (4864, 7785)	0.33 (0.25, 0.41)	13581 (10512, 16412)	0.53 (0.42, 0.64)
High SBP	327 (40, 700)	0.09 (0.01, 0.2)	589 (100, 1108)	0.05 (0.01, 0.10)	1057 (214, 1932)	0.05 (0.01, 0.09)	2773 (577, 4920)	0.14 (0.03, 0.26)	4131 (863, 7378)	0.16 (0.03, 0.29)
KD	689 (311, 1358)	0.21 (0.1, 0.42)	1378 (876, 2035)	0.13 (0.08, 0.19)	2453 (1670, 3209)	0.11 (0.08, 0.15)	5924 (3934, 7839)	0.31 (0.21, 0.42)	10640 (6948, 13909)	0.40 (0.27, 0.53)

**Abbreviations:** SDI: socio-demographic index; UI: uncertainty intervals; ASMR: age-standardized mortality rate; BMI: body-mass index; FPG: fasting plasma glucose; SBP: systolic blood pressure; KD: kidney dysfunction.

**Table 4:** Estimated annual percentage change in age-standardized rates of deaths and disability-adjusted life years attributable to metabolic risks for peripheral artery disease across different health systems (1990–2021).

Etiology	Minimal Health System		Limited Health System		Basic Health System		Advanced Health System	
-	EAPC from 1990 to 2021 of ASMR (95% CI)	EAPC from 1990 to 2021 of ASDR (95% CI)	EAPC from 1990 to 2021 of ASMR (95% CI)	EAPC from 1990 to 2021 of ASDR (95% CI)	EAPC from 1990 to 2021 of ASMR (95% CI)	EAPC from 1990 to 2021 of ASDR (95% CI)	EAPC from 1990 to 2021 of ASMR (95% CI)	EAPC from 1990 to 2021 of ASDR (95% CI)
Metabolic risks	2.12 (1.98, 2.25)	1.76 (1.63, 1.88)	1.83 (1.70, 1.96)	1.11 (1.07, 1.15)	0.09 (-0.02, 0.21)	0.07 (-0.01, 0.15)	-0.87 (-1.07, -0.67)	-0.78 (-0.94, -0.62)
High BMI	3.55 (3.22, 3.88)	3.08 (2.78, 3.38)	3.49 (3.39, 3.58)	3.17 (3.15, 3.2)	1.66 (1.53, 1.8)	1.88 (1.82, 1.95)	-0.44 (-0.67, -0.22)	-0.27 (-0.46, -0.09)
High FPG	3.09 (2.98, 3.2)	2.92 (2.82, 3.02)	2.71 (2.6, 2.82)	2.03 (1.99, 2.07)	0.51 (0.39, 0.63)	0.63 (0.48, 0.78)	0.79 (0.47, 1.11)	0.85 (0.60, 1.10)
High SBP	1.75 (1.65, 1.86)	1.32 (1.22, 1.41)	1.72 (1.63, 1.81)	0.9 (0.88, 0.92)	0.08 (-0.05, 0.21)	0.07 (-0.03, 0.16)	-1.88 (-2.08, -1.68)	-1.85 (-2.02, -1.68)
KD	1.72 (1.56, 1.87)	1.28 (1.14, 1.43)	1.4 (1.26, 1.54)	0.63 (0.59, 0.67)	-0.17 (-0.28, -0.07)	-0.27 (-0.34, -0.2)	-1.46 (-1.63, -1.28)	-1.39 (-1.53, -1.24)

**Abbreviations:** EAPC: estimated annual percentage change; CI: confidence interval; ASMR: age-standardized mortality rate; ASDR: age-standardized disability-adjusted life years rate; BMI: body-mass index; FPG: fasting plasma glucose; SBP: systolic blood pressure; KD: kidney dysfunction.

**Table 5:** Estimated annual percentage change in age-standardized rates of deaths and disability-adjusted life years attributable to metabolic risks for peripheral artery disease across different income regions (1990–2021).

Etiology	World Bank Low Income		World Bank Lower Middle Income		World Bank Upper Middle Income		World Bank High Income	
-	EAPC from 1990 to 2021 of ASMR (95% CI)	EAPC from 1990 to 2021 of ASDR (95% CI)	EAPC from 1990 to 2021 of ASMR (95% CI)	EAPC from 1990 to 2021 of ASDR (95% CI)	EAPC from 1990 to 2021 of ASMR (95% CI)	EAPC from 1990 to 2021 of ASDR (95% CI)	EAPC from 1990 to 2021 of ASMR (95% CI)	EAPC from 1990 to 2021 of ASDR (95% CI)
Metabolic risks	1.73 (1.6, 1.86)	1.32 (1.22, 1.43)	0.35 (0.18, 0.53)	0.44 (0.39, 0.49)	-2.30 (-2.48, -2.12)	-1.51 (-1.66, -1.36)	-0.55 (-0.76, -0.33)	-0.49 (-0.63, -0.35)
High BMI	3.35 (3.07, 3.63)	2.85 (2.62, 3.09)	0.33 (0.14, 0.52)	0.86 (0.76, 0.95)	-1.29 (-1.51, -1.07)	-0.36 (-0.54, -0.18)	-0.34 (-0.57, -0.12)	-0.23 (-0.39, -0.06)
High FPG	2.46 (2.34, 2.58)	2.25 (2.17, 2.33)	1.78 (1.66, 1.90)	1.74 (1.70, 1.79)	-0.78 (-0.96, -0.59)	-0.19 (-0.38, -0.01)	0.97 (0.62, 1.33)	0.97 (0.70, 1.24)
High SBP	1.57 (1.47, 1.66)	1.09 (1.02, 1.16)	-0.10 (-0.26, 0.07)	-0.01 (-0.06, 0.04)	-2.90 (-3.10, -2.69)	-2.00 (-2.18, -1.82)	-1.65 (-1.85, -1.46)	-1.67 (-1.81, -1.53)
KD	1.34 (1.2, 1.48)	0.85 (0.74, 0.97)	-0.16 (-0.35, 0.04)	-0.09 (-0.15, -0.03)	-2.90 (-3.08, -2.71)	-2.07 (-2.21, -1.93)	-1.13 (-1.30, -0.96)	-1.08 (-1.20, -0.97)

**Abbreviations:** EAPC: estimated annual percentage change; CI: confidence interval; ASMR: age-standardized mortality rate; ASDR: age-standardized disability-adjusted life years rate; BMI: body-mass index; FPG: fasting plasma glucose; SBP: systolic blood pressure; KD: kidney dysfunction.

**Table 6:** Estimated annual percentage change in age-standardized rates of deaths and disability-adjusted life years attributable to metabolic risks for peripheral artery disease across different SDI regions (1990–2021).

Etiology	Low SDI		Low-middle SDI		Middle SDI		High-middle SDI		High SDI	
-	EAPC from 1990 to 2021 of ASMR (95% CI)	EAPC from 1990 to 2021 of ASDR (95% CI)	EAPC from 1990 to 2021 of ASMR (95% CI)	EAPC from 1990 to 2021 of ASDR (95% CI)	EAPC from 1990 to 2021 of ASMR (95% CI)	EAPC from 1990 to 2021 of ASDR (95% CI)	EAPC from 1990 to 2021 of ASMR (95% CI)	EAPC from 1990 to 2021 of ASDR (95% CI)	EAPC from 1990 to 2021 of ASMR (95% CI)	EAPC from 1990 to 2021 of ASDR (95% CI)
Metabolic risks	1.81 (1.61, 2)	1.33 (1.22, 1.44)	2.05 (1.97, 2.14)	1.28 (1.24, 1.32)	-0.07 (-0.15, 0.01)	0 (-0.07, 0.06)	-2.21 (-2.38, -2.05)	-1.83 (-2.00, -1.67)	-0.36 (-0.64, -0.09)	-0.32 (-0.50, -0.13)
High BMI	3.23 (2.97, 3.49)	2.85 (2.67, 3.03)	3.57 (3.49, 3.66)	3.01 (2.97, 3.05)	1.50 (1.40, 1.61)	1.76 (1.7, 1.82)	-1.31 (-1.49, -1.14)	-0.92 (-1.09, -0.75)	-0.31 (-0.59, -0.03)	-0.16 (-0.35, 0.03)
High FPG	2.65 (2.49, 2.81)	2.33 (2.25, 2.42)	2.99 (2.92, 3.07)	2.31 (2.27, 2.36)	0.31 (0.23, 0.4)	0.54 (0.44, 0.64)	-0.88 (-1.11, -0.66)	-0.53 (-0.75, -0.31)	1.37 (0.94, 1.8)	1.31 (0.99, 1.64)
High SBP	1.60 (1.46, 1.75)	1.07 (1.01, 1.13)	1.85 (1.79, 1.92)	0.99 (0.96, 1.02)	-0.08 (-0.18, 0.02)	-0.04 (-0.12, 0.04)	-2.88 (-3.05, -2.7)	-2.47 (-2.64, -2.29)	-1.54 (-1.79, -1.28)	-1.57 (-1.73, -1.4)
KD	1.40 (1.18, 1.62)	0.85 (0.72, 0.97)	1.60 (1.51, 1.70)	0.77 (0.74, 0.81)	-0.31 (-0.39, -0.24)	-0.32 (-0.38, -0.27)	-2.68 (-2.84, -2.52)	-2.33 (-2.48, -2.18)	-1.02 (-1.25, -0.8)	-0.97 (-1.12, -0.83)

**Abbreviations:** SDI: socio-demographic index; EAPC: estimated annual percentage change; CI: confidence interval; ASMR: age-standardized mortality rate; ASDR: age-standardized disability-adjusted life years rate; BMI: body-mass index; FPG: fasting plasma glucose; SBP: systolic blood pressure; KD: kidney dysfunction.

## Data Availability

Publicly available datasets were analyzed in this study. This data can be found here: https://ghdx.healthdata.org/gbd-2021/sources.

## LIST OF ABBREVIATIONS

ASMR: Age-Standardized Mortality Rates
ASDR: Age-Standardized Disability-Adjusted Life Year Rates
ABI: Ankle-brachial Index
ACR: Albumin to Creatinine Ratio
ASR: Age-Standardized Rates
BMI: Body Mass Index
CI: Confidence Intervals
DALYs: Disability-Adjusted Life Years
EAPC: Estimated Annual Percentage Changes
FPG: Fasting Plasma Glucose
GBD: Global Burden of Disease
ICD: International Classification of Diseases
IHME: Institute for Health Metrics and Evaluation
KD: Kidney Dysfunction
LOESS: Locally Estimated Scatterplot Smoothing
PAD: Peripheral Arterial Disease
SBP: Systolic Blood Pressure
SDI: Socio-Demographic Index
TMREL: Theoretical Minimum Risk Exposure Level
UI: Uncertainty Intervals
YLLs: Years of Life Lost
YLDs: Years Lived with Disability
